# *Simulcast*: a case study in the establishment of a virtual community of simulation practice

**DOI:** 10.1186/s41077-020-00122-4

**Published:** 2020-05-27

**Authors:** Benjamin Symon, Jesse Spurr, Victoria Brazil

**Affiliations:** 1 Simulation Training Optimising Resuscitation for Kids (STORK), Queensland Children’s Hospital, Brisbane, Australia; 2 School of Clinical Medicine, University of Queensland, Brisbane, Australia; 3 Intensive Care Unit, Redcliffe Hospital, Redcliffe, Australia; 4 Faculty of Health Sciences and Medicine, Bond University, Gold Coast, Australia

## Abstract

Virtual Communities of Practice (vCoP) is a nascent approach to professional development for simulation educators (Thoma et al., Simul Healthc. 2018;13(2):124-30). vCoPs overcome geographic barriers to accessing expertise and professional networks and may promote ‘democratisation’ of voices in the simulation community. However, the optimal process for creating, nurturing and joining vCoPs in healthcare simulation is not well understood.

We report on the establishment of our healthcare simulation hybrid podcast/blog—*Simulcast* (www.simulationpodcast.com)—utilising the conceptual framework of Wenger’s three dimensions of Communities of Practice. In exploring these dimensions—joint enterprise, mutual engagement and shared repertoire—we hope to contextualise vCoP within professional development approaches for simulation faculty and invite readers to engage with our existing community.

## Virtual CoPs

Virtual Communities of Practice (vCoP) has been described as a virtual environment where *people can share a concern, a set of problems, or a passion about a topic and can deepen their knowledge and expertise in this area by interacting on an ongoing basis* [1, 2]. Thoma et al. emphasise sociocultural aspects of participation within a vCoP—*Learning in a CoP is a collaborative and social process with thinking that is situated in a cultural context* [3].

Not every collection of individuals engaged in learning is necessarily a community of practice [4], and Wenger describes three key features of CoP as follows:

*What it is about: its joint enterprise as understood and continually renegotiated by its members.*

*How it functions: the relationships of mutual engagement that bind members together into a social entity.*

*What capability it has produced: the shared repertoire of communal resources (routines, sensibilities, artifacts, vocabulary, styles, etc.) that members have developed over time* [5].

Connecting simulation practitioners with resources, experts, academics and each other remain persistently challenging. VCoP has been promoted as potential lifelines of professional development within the simulation education community, in part based on the success of parallel communities in emergency medicine [6–8] and health professional education [9]. A number of simulation vCoP exist, with variable origins from educational, professional and commercial institutions, diverse curricular emphases and several with barriers to entry such as subscription models or face to face course enrolment. Palaganas et al. describe a multimodal approach to creating an interprofessional simulation vCoP focused on developing feedback skills [10].

Wenger’s theories have an *emphasis on active apprenticeship and legitimate peripheral participation as a model for ‘becoming’ a central member of an established community* [5], while others note that educationalist interpretation of Wenger’s descriptions has trended toward an ‘instrumental’ model aiming for ‘deliberate group cultivation’ [4]. Within this article, we reference both legitimate peripheral participation and instrumental aspects of Wenger’s theory, with more emphasis on the latter.

## Simulcast

*Simulcast* was established in July 2016, as a monthly podcast focused on professional development for simulation educators. Early podcast episodes focused on topics such as simulation debriefing, psychological safety and scenario design, with a format of host interviewing an academic or expert in the field. In parallel, we established a monthly journal club, with a format based on the Academic Life in Emergency Medicine (ALiEM) ‘MeDIC’ series [8]—a written case study posted on the website, promoted on Twitter, with comments invited from readers and then ‘wrapped’ at the end of the month in a summary with expert commentary. The interest and engagement in the journal club prompted the addition of a monthly journal club podcast, reviewing the discussion from the previous month, drawing learning from the expert commentary and previewing the feature article for the next month. These episodes included a commentary on three other recent articles from the published simulation literature.

Since 2018, *Simulcast* and the open access journal *Advances in Simulation* have collaborated to produce bimonthly podcasts discussing an article nominated by the journal Editor in Chief, including a simulcast producer, article author and additional discussant with expertise in the relevant area.

Simulcast has also partnered with conferences relevant to the simulation community—Australasian Simulation Congress (ASC), International Meeting for Simulation in Healthcare (IMSH) and Society in Europe Applied to Simulation in Medicine (SESAM)—to ‘cover’ the events, similar to a mainstream media approach.

The technical elements of *Simulcast* production are managed solely by the producers. The website is a WordPress (https://wordpress.com/) blog-based platform, hosted on a VPN by Bluehost (https://www.bluehost.com/). When guests and hosts are connecting remotely, the podcast audio is recorded using Zencastr (https://zencastr.com/), then edited using either GarageBand (https://www.apple.com/au/ios/garageband/) or Hindenburg (https://hindenburg.com/) and optimised using Auphonic (https://auphonic.com/). The podcasts are then published to the hosting platform Podbean (https://www.podbean.com/) and distributed to iTunes Podcasts and Spotify (https://www.spotify.com/au/) via RSS feed. There are many alternatives for website build, hosting, recording editing and distribution, and these mentioned are solely for demonstration and transparency of our process.

Our resources and podcasts are free, and financial and other costs are borne by the producers.

At the time of writing, the site hosts an archive of over 80 podcasts and more than 30 online journal club discussions, with over 80k podcast downloads and 100k site hits.

## Joint enterprise—*why*?

Our mission is articulated on the *Simulcast* home page—‘to bridge the gap between the simulation researcher, working in the academic centres, and the clinician delivering education at the point-of-care.’ This has provided an anchoring set of values for decisions regarding production and style or ‘brand’, relationships and subject focus.

The production and style have been shaped by the feedback from the community—suggesting guests, topics and technical enhancements and also reflecting on how changes have affected their contribution to the blog. Production quality is a priority but within the time and financial constraints of being impartial and free. Relationships have been considered in terms of shared mission, values and brand associations, as opposed to commercial partnerships. Profiling technology and industry advances without the encumbrance of financial relationships allows credible critique of new products and techniques.

The original mission was born of desire for translation from academic to practical, a ‘bridge’, with a flow of ideas into the community for reflection and discussion. With active participation of readers and listeners, this bridge has become increasingly bidirectional—with space created for discussion between researchers and authors and health professional educators at the clinical coalface. As described by Wenger, a community of practice continually renegotiates its joint enterprise.

## Mutual engagement—*who*?

The CoP starts with the Simulcast producers. Vic is an emergency physician, academic and educator whose simulation experience includes undergraduate education, translational simulation, high performance team training and faculty development in health professional education. Her interest in social media was encouraged by participation in the emergency medicine vCoP. Ben is a paediatric emergency physician and simulation consultant with experience delivering translational and educational simulation in regional Queensland and a degree in visual arts in animation. He had previously established an international simulation journal club distributed via email. Jesse is an intensive care nurse with experience in nursing, medical and corporate health professional education and translational and educational simulation in settings throughout hospitals, public spaces and primary healthcare facilities. Jesse also had prior interest and experience in blog and podcast production and research interest in vCoP in health education [11].

Although seeking broad connection and reach, our strategy has been *don*’*t confuse traffic with audience*, *and audience with community*. In aspiring to the description of a vCoP, we consider our engagement as a *network*—with academics, journals, book editors, faculty development providers, events, industry and ‘coalface’ simulation educators—rather than a repository of information to be disseminated. This has required strategies to cater for network members with a broad range of motivations, learning needs and social media proficiency.

Through interviews, book reviews, infographics and journal clubs featuring new publications, we have worked with academics to translate their knowledge to the broader simulation community. Fulfilling the promise of democratisation, our vCoP sees lead authors contribute to journal club to thank and reflect on the comments of those ‘legitimate peripheral participants’.

Collaboration with *Advances in Simulation* has connected our social media reach with the journal’s academic credibility and reputation. We have drawn on our own professional networks for guest appearances, advice and inspiration and added to those networks during interviews and through developing content for *Simulcast*. Our existing collaborations are not exclusive, and we aspire to more.

In considering *Simulcast* to be more than a broadcast, we have intentional social media strategies to encourage bidirectional engagement and ‘deliberate group cultivation’ [4]. Applying Michael Hyatt’s framework of ‘home base, embassies and listening posts’ for online presence [12], the Simulcast website is a ‘home base’ for engagement, with established ‘embassies’ in Twitter, Facebook, Instagram and LinkedIn to connect with different craft groups within the simulation community. The *Simulcast* team also contribute to ‘outposts’—other blogs and podcasts in which we are guests, but also ambassadors for *Simulcast*, in a strategy inspired by ALiEM [13].

## Shared repertoire—*how*? *And what*?

Although rising in popularity, social media-based resources for health professional education require new (and old) processes to ensure quality and credibility [14], while realising their potential for engaging formats and easy accessibility.

*Simulcast* originated as a podcast, as part of a global trend in popularity of this media format. There is broad appeal in the convenience of ‘short form’ media accessed on a phone, consumed in a drive or train ride, combined with the neurochemical triggers of powerful audio storytelling [15]. Podcasting, and the broader use of social media, for educational purpose has become popular [16] and especially so in the emergency medicine and critical care disciplines [6, 7]. Our choice of conversational style and interview format provides contrasting views and perspectives. Cases and storytelling have enabled our experts and guests to articulate their philosophies and own practice revelations that did not fit in the confines of a scholarly publication (Table 1).

The *Simulcast* Journal Club is the primary pathway for bidirectional connection between our community and hosts via social media, blog comments or real-life interactions. In many ways, the journal club demonstrates key features of legitimate peripheral participation, with simulation enthusiasts and researchers having the opportunity to contribute to discussion between prominent world experts and relative novices in a social, relaxed format. Participants have the option to titrate their interaction based on comfort level and their perception of ‘membership’, moving from anonymous readers to enthusiastic contributors at a pace that suits their learning needs. The journal club enhances appeal through combining the monthly podcast, frequent infographic summaries of literature, case vignettes that form a serial, satirical melodrama when read in sequence and downloadable Ebook collections. Over 3 years, there has been a global reach—with contributions from South Africa, China, the UK, New Zealand, Australia, Canada and the USA. There have been over 700 blog post comments on the website with over 650 of these being active discussant contributions in journal clubs.

**Table 1:** Examples of shared repertoire

Examples of shared repertoire from the Simulcast vCoP		
Category	Learning	Example
Advances in simulation episodes	Interpretation and debate of new publication with translation of principles to clinical educators	Episode 75: Another Debriefing Course! Who benefits?
Podcast interview	Institutional connection and cross pollination of ideas	Episode 60: CMS and Simulcast Translational Simulation
Conference coverage	Previews, soundbites and summaries of learning from prominent simulation conferences	Episode 65: Ben & Vic at IMSH with KT Waxman, Komal Bajaj & James Leung
Journal club	Discussion of classic and new articles in simulation literature	November 2017: Sticks and Stones
Journal club eBook	Collection of journal club summaries and expert commentaries for each year	Simulcast Journal Club: The Second Year

## Lessons, questions and next steps for *Simulcast*

We have numeric analytics for downloads and website hits, but we have not performed any formal evaluation of *the impact* of *Simulcast*. Outcomes we expected have been consumption by individual podcast listeners, increased Altmetrics for journal articles featured on Simulcast and many new friends and professional colleagues. Less expected outcomes have included the use of Simulcast content in short courses and fellowship programs and integrated into academic curricula. Our online journal club has been embraced by IRL (‘in real life’) journal clubs for local institutions/simulation programs.

Our *scope* has evolved—our ability to deliver focused, in depth content is in tension with the diversity of practitioners we aim to include in the *Simulcast* vCoP. We recognise a bias toward debriefing and educational aspects of simulation in our content and know that we cannot provide a comprehensive scope that is fully reflective of the diversity within healthcare simulation.

Podcast *quality*—technical and educational—is a critical element in an era of overwhelming online possibilities. Our posts and podcast content are not peer reviewed, but we have appreciated some frank feedback from colleagues. Although technology has dramatically lowered the barriers to entry for podcasters—inexpensive hardware and intuitive software for recording and editing and a plethora of online advice [17]—high quality production can remain challenging. Our journey in pursuit of sound quality has required research, feedback and volume of experience.

*Establishing a safe container* for online learning has proved challenging. Despite promoting reflection and shared vulnerability, simulation educators remain as reticent as other learners when it comes to intellectual candour [18]. Deliberate strategies to build online psychological safety have included conscious role modelling, consistent enforcement of conversational boundaries and long form storytelling. Self-satire and humour, in particular, have been noted to defuse an implicit sense of hierarchy present in discussion subgroups.

Our *sustainability* is supported by a producer trio with different perspectives and skill sets, allowing workload sharing and diverse outputs. We note cautions from others who are active in promoting vCoPs in over-extending our capacity [19]. Our hosting costs may become more significant if our audience volume grows significantly. Although we support the #FOAMsim ethos of resources being free to consumers, we are not deluded that our production does not have a cost. Nor are we naïve to issues of intellectual property and the need for return on investment for commercial organisations that will limit boundless sharing and collaboration.

## Conclusion

We hope this review assists others considering establishing or joining a virtual community of practice and invite readers to participate as listeners, contributors or colleagues with *Simulcast*.

## Supplementary information

**Additional file 1.** Simulcast Infographic. An infographic depicting Simulcast through the lens of Wenger’s pillars of communities of practice.

## Data Availability

This article comprises primarily conceptual material regarding Simulcast as a virtual community of practice. As such there is minimal statistical data beyond our download and hit statistics, which can be provided by Jesse Spurr.

## Abbreviations

CoP: Communities of Practice
vCoP: Virtual Communities of Practice
VPN: Virtual private network
RSS: Really Simple Syndication
ALiEM: Academic Life in Emergency Medicine
MEdIC: Medical Education in Cases
IRL: In real life
#FOAMSim: Free Open Access Medical Education on Simulation
