# Supplementary figures and images for: Simulcast: a case study in the establishment of a virtual community of simulation practice
Source: Adv Simul (Lond). 2020 May 27;5:5. doi: 10.1186/s41077-020-00122-4 (PMC7251887; doi:10.1186/s41077-020-00122-4)

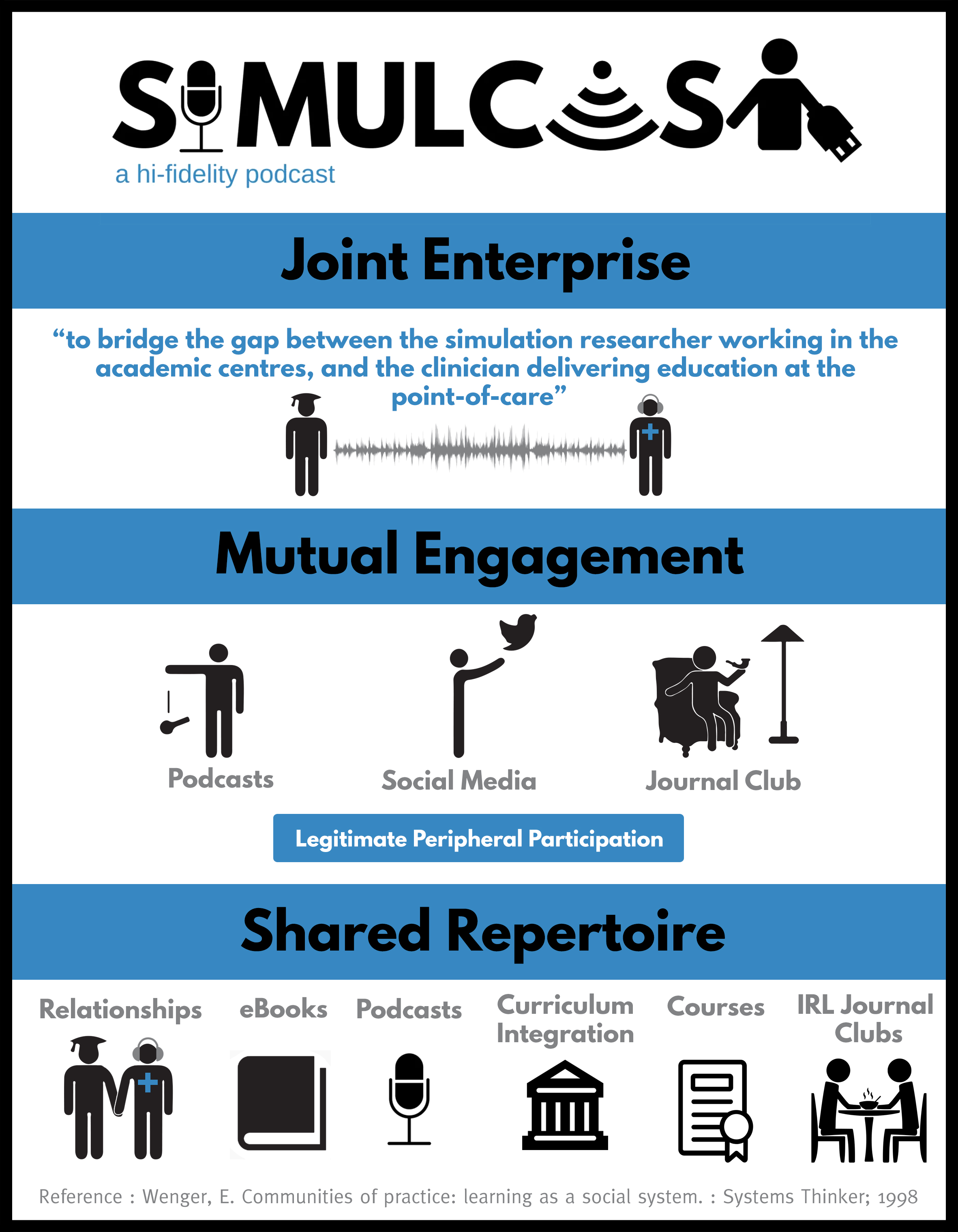

Supplement: Supplementary file 1 — Additional file 1. Simulcast Infographic. An infographic depicting Simulcast through the lens of Wenger’s pillars of communities of practice. [file 41077_2020_122_MOESM1_ESM.jpg]
